# Baseline Cardiac Biomarker Levels as Predictors of Cancer Risk in the MESA Cohort

**DOI:** 10.1016/j.jacadv.2025.101884

**Published:** 2025-06-16

**Authors:** Xinjiang Cai, Quinn White, W. Craig Johnson, Spencer L. Hansen, Zeina A. Dardari, Michael Blaha, Erin D. Michos, Joao AC. Lima, Christopher R. deFilippi, Matthew J. Budoff, Karol E. Watson, Robyn L. McClelland, Eric H. Yang

**Affiliations:** a Division of Cardiology, Department of Medicine, David Geffen School of Medicine at UCLA, Los Angeles, California, USA; b Department of Biostatistics, University of Washington School of Public Health, Seattle, Washington, USA; c Johns Hopkins Ciccarone Center for the Prevention of Cardiovascular Disease, Baltimore, Maryland, USA; d Division of Cardiology, Department of Medicine, Johns Hopkins School of Medicine, Baltimore, Maryland, USA; e Cardiovascular Medicine, Inova Heart and Vascular Institute, Falls Church, Virginia, USA; f Division of Cardiology, The Lundquist Institute at Harbor-UCLA Medical Center, Los Angeles, California, USA; g UCLA Cardio-Oncology Program, Division of Cardiology, Department of Medicine, David Geffen School of Medicine at UCLA, Los Angeles, California, USA

**Keywords:** biomarkers, colorectal cancer, lung cancer, outcomes, risk factor, risk prediction

## Abstract

**Background:**

Assessing the association between baseline levels of cardiac biomarkers and future cancer risk is critical to understand the cross talk between cardiovascular disease and cancer.

**Objectives:**

The authors aimed to determine the association between baseline levels of high-sensitivity cardiac troponin T (hs-cTnT) and N-terminal pro-B-type natriuretic peptide (NT-proBNP) with cancer risk in the prospective MESA (Multi-Ethnic Study of Atherosclerosis) cohort.

**Methods:**

We analyzed data from 6,244 MESA participants free of self-reported cancer and cardiovascular disease at baseline. Incident cancer was identified using International Classification of Diseases-9th Revision codes from hospitalizations. Cox proportional hazards models were employed to evaluate the associations of hs-cTnT and NT-proBNP with cancer risk. Likelihood ratio tests assessed whether these associations differed by race/ethnicity or sex.

**Results:**

The median age was 61.0 years, with 52.7% being female. Over a median follow-up period of 17.8 years, there were 820 incident cancer events, with an incidence rate of 91.2 cases per 10,000 person-years. Higher incidence rates for all cancers were generally associated with higher baseline hs-cTnT and NT-proBNP levels, especially in the highest quartiles. For all-cancer endpoints, the HRs of hs-cTnT and NT-proBNP, calculated based on the SDs for continuous covariates after standardization, were statistically significant in fully adjusted models (HR: 1.18; 95% CI: 1.09-1.27; *P* < 0.001; and HR: 2.41; 95% CI: 1.30-4.49; *P* = 0.006, respectively). Sex and race/ethnicity did not significantly affect any of these associations.

**Conclusions:**

In the MESA cohort, higher baseline levels of hs-cTnT and NT-proBNP predicted an increased risk of incident cancer, with no significant differences by race/ethnicity or sex.

Cardiovascular disease (CVD) and cancer are 2 major public health challenges worldwide.^1^^,^^2^ With an expanding aging population and advancements in early cancer detection and new treatment strategies, the number of cancer survivors is projected to continue to rise.^3^^,^^4^ Cancer and CVD share several common risk factors, including aging, smoking, diabetes mellitus, obesity, and sedentary lifestyle.^5^, ^6^, ^7^ Cancer survivors may have coexisting CVD and/or a high prevalence of cardiovascular risk factors before cancer diagnosis, which predisposes them to elevated cardiovascular toxicities from anticancer treatment.^5^^,^^8^, ^9^, ^10^, ^11^, ^12^ Cardiac biomarkers, such as high-sensitivity cardiac troponin T (hs-cTnT) and N-terminal pro-B-type natriuretic peptide (NT-proBNP) have been used for risk stratification of cardiovascular toxicity before initiating anticancer therapy and for monitoring cardiovascular complications during treatment.^13^, ^14^, ^15^, ^16^ Elevated levels of these cardiac biomarkers and cardiac dysfunction are frequently associated with increased cardiac events and mortality in cancer patients.^17^, ^18^, ^19^, ^20^

Recent animal studies have made significant advancements by demonstrating that myocardial infarction, heart failure, and early cardiac remodeling, even in the absence of heart failure, can accelerate cancer growth and metastasis.^21^, ^22^, ^23^ Collectively, these findings highlight the cross talk between CVD and cancer and identify potential signaling pathways and secretory factors that facilitate cross-disease communication.^5^^,^^6^ However, the remaining question is whether baseline levels of cardiac biomarkers, traditionally used to identify individuals at risk for structural cardiac abnormalities and cardiac dysfunction,^24^^,^^25^ are associated with increased cancer incidence. This was addressed by 2 elegant studies^22^^,^^26^ involving 2 distinct cohort populations—the PREVEND (Prevention of Renal and Vascular End-Stage Disease) study and the FHS (Framingham Heart Study). In the PREVEND cohort from Groningen, the Netherlands, elevated hs-cTnT and NT-proBNP levels were associated with an increased risk of incident cancer.^22^ In contrast, while traditional CVD risk factors in both the FHS and PREVEND studies were predictive of higher cancer risk, elevated natriuretic peptide levels, but not high-sensitivity troponin levels, were associated with higher cancer incidence.^26^ These findings suggest a possible link between baseline cardiac biomarkers and cancer, warranting further investigation into the clinical relevance of subclinical cardiac injury and remodeling in cancer risk assessment.

The MESA (Multi-Ethnic Study of Atherosclerosis) is a prospective cohort study investigating the progression of subclinical CVD across 4 racial/ethnic groups including non-Hispanic White, Black, Hispanic/Latino, and Chinese participants from 6 urban study centers.^27^^,^^28^ In this report, we aimed to determine the association of baseline levels of biomarkers, hs-cTnT and NT-proBNP, with risk of developing incident cancer among individuals initially free of both clinical CVD and cancer in a multiethnic, geographically diverse cohort. In addition, we sought to examine whether race/ethnicity and sex affect these associations.

## Methods

### Study population

The MESA study is a longitudinal cohort study that enrolled 6,814 men and women aged 45 to 84 years with no clinical history of CVD between 2000 and 2002 (Exam 1). MESA participants from 4 racial/ethnic groups (non-Hispanic White, Black, Hispanic/Latino, and Chinese) were recruited across 6 field centers in the United States.^27^^,^^29^ Subsequently, participants underwent further clinical evaluation to determine the characteristics and risk factors associated with the progression of subclinical CVD to clinically overt CVD over subsequent in-person follow-up examinations until Exam 6 (2016-2018). Plasma samples were also collected for the measurement of biomarkers and other relevant parameters.

Informed consent was obtained from all participants in the MESA study. The study protocol was approved by the Institutional Review Boards of all participating centers. Comprehensive details regarding the study protocol are available on the MESA website (https://www.mesa-nhlbi.org).

### Baseline characteristics and biomarker assays

Demographic information, CVD risk factors, and clinical characteristics were collected as previously reported,^27^^,^^30^ including covariates such as age, sex, race/ethnicity, education level, health insurance status, use of antihypertensive medications, systolic blood pressure, lipid levels, statin use, renal function, physical activity, and dietary patterns at Exam 1. Details on baseline characteristics and biomarker assays are available in Supplemental Methods.

### Outcome definitions and cancer events

The median follow-up period for MESA participants was 17.8 years (Q1-Q3 12.8-18.5 years). During the follow-up period, in addition to baseline and 6 follow-up Exams, the MESA cohort participants or their family members were contacted by telephone every 9 to 12 months.^27^^,^^31^ Telephone interviewers collected information on interim hospital admissions, CVD diagnoses and procedures, and deaths. Additional details were gathered through cohort clinic visits, participant-initiated contacts, and medical record data abstraction. Diagnosis codes from the International Classification of Diseases-9th Revision (ICD-9) were retrieved from hospitalization records, and cancer-related ICD-9 codes were extracted and recoded into specific cancer entities.^32^ The term “All Cancers” refers to all registered and defined cancer type groups, excluding nonmelanoma skin cancers, with the MESA cohort. Female-specific cancer endpoint is comprised of ovarian, breast, and uterine cancers.

### Statistical analysis

Baseline characteristics are reported as median values with 25th-75th percentiles (Q1-Q3) for continuous variables or frequencies (%) for categorical variables. Baseline characteristics are reported by incident cancer status and were compared using Wilcoxon rank sum test and chi-squared test for continuous variables and categorical variables, respectively. We examined hs-cTnT and NT-proBNP levels as predictor variables using 2 methods, as previously reported^25^^,^^33^: 1) NT-proBNP concentrations were divided into 4 quartiles, while hs-cTnT concentrations were categorized into 5 predefined groups, including one category below the limit of detection (LOD) and 4 quantiles for the remaining measured values; and 2) continuous log-transformed hs-cTnT and NT-proBNP levels were included in Cox proportional hazards models, where the log transformation was performed due to the highly skewed distribution of the original values.

Cumulative incidence rates for all cancers, specific cancer subtypes (lung cancer, colorectal cancer, and breast cancer), and sex-specific cancers (prostate cancer for men and ovarian, uterine, and breast cancer for women) were calculated and presented per 1,000 person-years. The incidence rates were stratified based on baseline levels of hs-cTnT and NT-proBNP as previously reported. For hs-cTnT levels,^25^ the categories were <3.0 ng/L (LOD), 3.0 to 4.25 ng/L, 4.26 to 5.87 ng/L, 5.88 to 8.80 ng/L, and ≥8.81 ng/L. For NT-proBNP levels,^25^ participants were divided into 4 quartiles.

We fit a Cox proportional hazards model to study the association between baseline characteristics and the hazard of cancer. The baseline characteristics considered were sex, systolic blood pressure, diabetes, body mass index, total cholesterol, high-density lipoprotein cholesterol, age, smoking status, pack years of cigarette smoking, study site, race/ethnicity, diet, education, moderate to vigorous physical activity, statins, and health insurance status. Results are presented as HR with 95% CI.

Separate Cox proportional hazards models were utilized to study the association between each primary predictor and the cancer endpoint of interest. For each primary predictor and cancer endpoint, we fit both a minimally and fully adjusted model. Minimally adjusted models were adjusted for age, race/ethnicity, study site, sex, body mass index, education, and health insurance status. Fully adjusted models included all the covariates in the minimally adjusted as well as diabetes, hypertension medication, systolic blood pressure, total cholesterol, high-density lipoprotein cholesterol, smoking status, pack years of cigarette smoking, statins, and estimated glomerular filtration rate. For the colorectal endpoint, diet and moderate to vigorous exercise were also included for the fully adjusted models, and for the breast and female-specific endpoints, female-specific covariates including menopausal stage, hormone replacement therapy, and age at first birth (age <21 years; age ≥21 to <25 years; age ≥25 years; no children) were also included.

To test if the association of any of the primary predictors differed by race/ethnicity, we fit each of the fully adjusted models including the relevant interaction term and compared the model with the interaction term to that without it with a likelihood ratio test. A Bonferroni-adjusted threshold was used for testing the interaction terms.

For all the Cox proportional hazards models considered, a cause-specific approach to competing events was taken, where an individual was censored for a cancer outcome at the time any other cancer type was diagnosed. Additionally, all continuous covariates were standardized prior to model fitting, and the proportionality of hazards assumption was assessed for each model via testing the correlation between the Schoenfeld residuals and time. Because of the standardization, the HR of any continuous covariate is the HR per SD in that covariate. Log linearity was verified for each primary predictor via inspection of the martingale residuals. We considered a *P* value <0.05 statistically significant, and all statistical analyses were conducted using R version 4.4.0 (R Core Team [2024], R Foundation for Statistical Computing).

## Results

### Study population and baseline characteristics

Among 6,244 participants in the MESA study population free of CVD and self-reported cancer at baseline at Exam 1, 6,035 and 6,043 participants were included in the analyses for the association of hs-cTnT and NT-proBNP, respectively, with incident cancer. Participants with missing levels of hs-cTnT, NT-proBNP, or covariates were excluded, as illustrated in Supplemental Figure 1. Also excluded were 4 male participants who were diagnosed with breast cancer and 1 female participant who was recorded to have prostate cancer. The baseline demographic characteristics, cardiovascular risk factors, and clinical features of 6,244 participants included in the MESA study and 3,290 female participants are presented in Tables 1 and 2. The median age was 61.0 years (Q1-Q3: 53.0-70.0 years) and 52.7% were female.

**Table 1:** Baseline Characteristics

	Total Participants (N = 6,244)	Incident Cancer (n = 820)	No Incident Cancer (n = 5,424)
Age at exam 1 (y)	61.0 (53.0, 70.0)	66.0 (57.0, 73.0)	61.0 (52.0, 69.0)
Race/ethnicity			
White	2,284 (36.6%)	362 (44.1%)	1,922 (35.4%)
Black	1,764 (28.3%)	255 (31.1%)	1,509 (27.8%)
Hispanic/Latino	1,417 (22.7%)	137 (16.7%)	1,280 (23.6%)
Chinese	779 (12.5%)	66 (8.0%)	713 (13.1%)
Site			
WFU	945 (15.1%)	167 (20.4%)	778 (14.3%)
COL	1,032 (16.5%)	132 (16.1%)	900 (16.6%)
JHU	985 (15.8%)	116 (14.1%)	869 (16.0%)
UMN	976 (15.6%)	147 (17.9%)	829 (15.3%)
NWU	1,058 (16.9%)	141 (17.2%)	917 (16.9%)
UCLA	1,248 (20.0%)	117 (14.3%)	1,131 (20.9%)
Sex			
Male	2,954 (47.3%)	469 (57.2%)	2,485 (45.8%)
Female	3,290 (52.7%)	351 (42.8%)	2,939 (54.2%)
Body mass index (kg/m^2^)	27.6 (24.6, 31.2)	27.7 (24.6, 31.4)	27.6 (24.5, 31.2)
Education			
Less than high school	1,158 (18.6%)	131 (16.0%)	1,027 (19.0%)
High school graduate	2,894 (46.5%)	396 (48.3%)	2,498 (46.2%)
College	1,071 (17.2%)	138 (16.8%)	933 (17.3%)
Graduate school	1,102 (17.7%)	155 (18.9%)	947 (17.5%)
No health insurance	584 (9.4%)	41 (5.0%)	543 (10.0%)
Diabetes	788 (12.7%)	109 (13.3%)	679 (12.6%)
Used hypertension medication	2,287 (36.6%)	342 (41.7%)	1,945 (35.9%)
Seated systolic blood pressure (mm Hg)	123.5 (111.0, 139.5)	125.5 (113.0, 141.0)	123.0 (110.5, 139.5)
Total cholesterol (mg/dL)	192.0 (171.0, 216.0)	190.0 (168.0, 214.0)	193.0 (171.0, 216.0)
HDL cholesterol (mg/dL)	48.0 (40.0, 59.0)	47.0 (40.0, 57.0)	48.0 (40.0, 59.0)
Smoking status			
Former	2,225 (35.7%)	333 (40.6%)	1,892 (35.0%)
Never	3,166 (50.9%)	339 (41.3%)	2,827 (52.3%)
Current	835 (13.4%)	148 (18.0%)	687 (12.7%)
Exam 1 pack-years of cigarette smoking	0.0 (0.0, 15.0)	4.5 (0.0, 26.4)	0.0 (0.0, 13.5)
Used statins	908.0 (14.6%)	133 (16.3%)	775 (14.3%)
CKD-Epi eGFR based on exam 1 scale Cr	78.3 (67.4, 89.3)	75.2 (65.4, 86.9)	78.8 (67.9, 89.5)
Moderate and vigorous physical activity total (MET-min/wk Monday-Sunday)	4,047.5 (1,980.0, 7,633.1)	4,040.0 (2,010.0, 7,293.8)	4,050.0 (1,980.0, 7,680.0)
Cardiovascular health factor: diet			
Not poor	2,411 (40.3%)	303 (38.5%)	2,108 (40.5%)
Poor	3,575 (59.7%)	484 (61.5%)	3,091 (59.5%)

Values are median (Q1, Q3) or n (%).

CKD = chronic kidney disease; COL = Columbia University Field Center; eGFR = estimated glomerular filtration rate; HDL = high-density lipoprotein; JHU = Johns Hopkins University Field Center; NWU = Northwestern University Field Center; UCLA = University of California, Los Angeles Field Center; UMN = University of Minnesota Field Center; WFU = Wake Forest University Field Center.

**Table 2:** Female-Specific Baseline Covariates

	Total (N = 3,290)	Incident Cancer (n = 351)	No Incident Cancer (n = 2,939)
Menopause			
Postmenopausal	2,634 (82.4%)	302 (87.0%)	2,332 (81.8%)
In menopause	244 (7.6%)	18 (5.2%)	226 (7.9%)
Premenopausal	320 (10.0%)	27 (7.8%)	293 (10.3%)
Used hormone replacement therapy			
Yes	1,501 (50.5%)	172 (52.4%)	1,329 (50.2%)
No	1,473 (49.5%)	156 (47.6%)	1,317 (49.8%)
Age at first birth			
<21 y	904 (31.4%)	90 (30.1%)	814 (31.5%)
≥21 to <25	831 (28.8%)	98 (32.8%)	733 (28.4%)
≥25	968 (33.6%)	93 (31.1%)	875 (33.9%)
No children	180 (6.2%)	18 (6.0%)	162 (6.3%)

Values are n (%).

**Figure 1:**
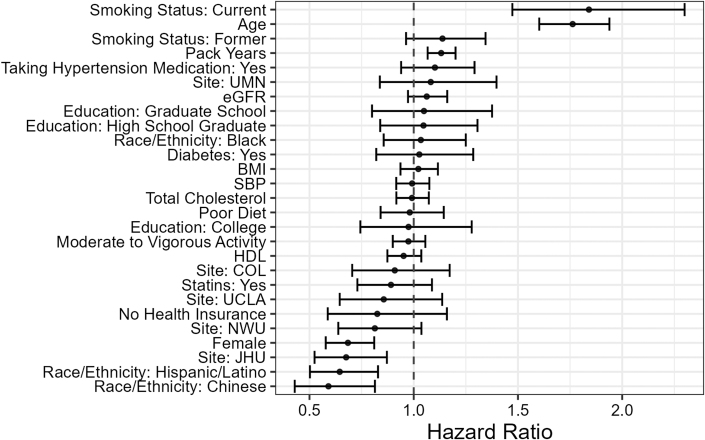
HRs With 95% CIs Predicting Cancer Risk in the Fully Adjusted Model The reference category for site is Wake Forest University, for smoking status is never smoked, for education it is less than high school, and for race/ethnicity it is White. BMI = body mass index; COL = Columbia University Field Center; eGFR = estimated glomerular filtration rate; HDL = high-density lipoprotein; JHU = Johns Hopkins University Field Center; NWU = Northwestern University Field Center; SBP = systolic blood pressure; UCLA = University of California, Los Angeles Field Center; UMN = University of Minnesota Field Center.

Over a median follow-up period of 17.8 years (Q1-Q3: 12.8-18.5 years), there were 820 new first cancer events, with an incidence rate of 91.17 cases per 10,000 person-years (Table 3) and a median time to cancer detection of 7.95 years (Q1-Q3: 4.31-12.54). As shown in Figure 1, smoking, age, sex, and race/ethnicity were significant predictors of incident cancer, as expected.

**Table 3:** Incidence Rates by Cancer Subtype

Cancer	Incidence (per 10,000 Person-Years)
All cancer	91.17 (n = 820)
Prostate	37.96 (n = 157)
Female specific	19.76 (n = 96)
Lung	12.9 (n = 116)
Breast	12.55 (n = 61)
Colorectal	8.01 (n = 72)

### Incidence rate of cancer subtypes and stratification by hs-cTnT and NT-proBNP

Among all cancer subtypes, prostate cancer, which was also the most common cancer among male participants, had the highest incidence rate across all participants at 37.96 cases per 10,000 person-years. This was followed by the female-specific endpoint comprised of ovarian, breast, and uterine cancers, which an incidence rate of 19.76 cases per 10,000 person-years (Table 3).

**Figure 2:**
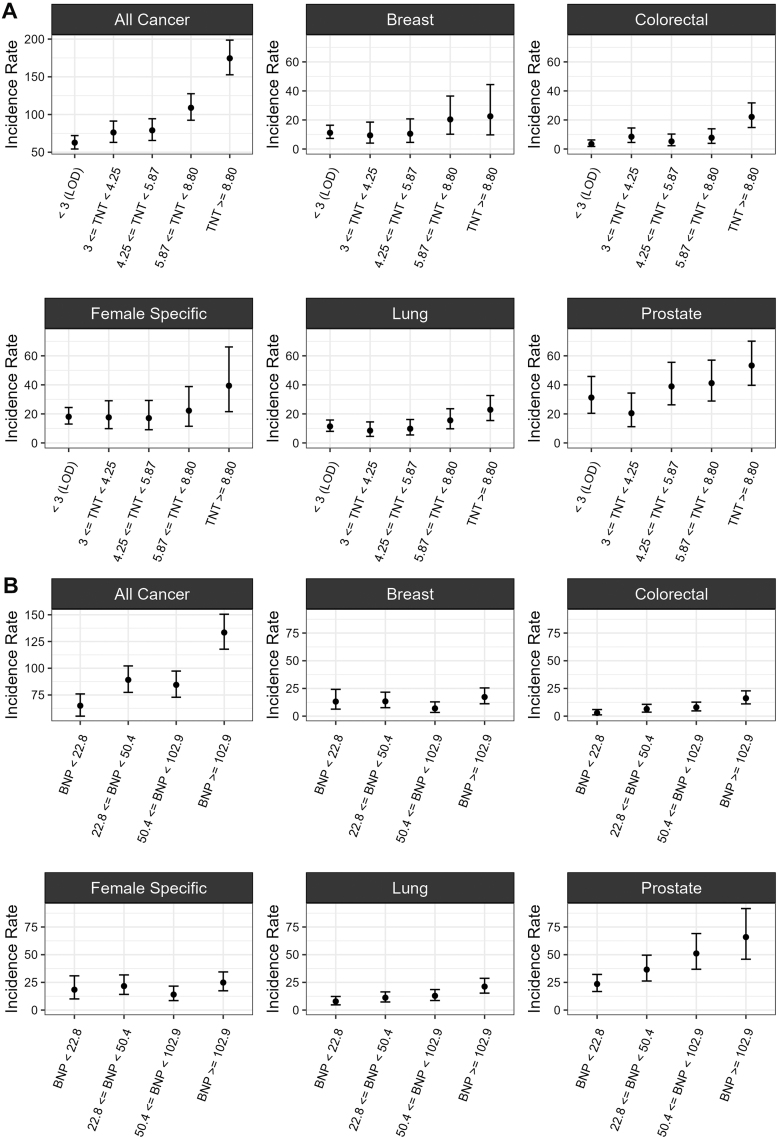
Incidence Rates With 95% CIs of All Cancers and Cancer Subtypes Stratified by hs-cTnT and NT-proBNP Levels (A) hs-cTnT (TNT) and (B) NT-proBNP (BNP). Incidence rates were calculated as per 10,000 person-years. The hs-cTnT strata were defined according to Seliger et al,^25^ while the NT-proBNP strata were based on its quartile distribution. BNP = N-terminal pro-B-type natriuretic peptide; LOD = limit of detection; TNT = high-sensitivity cardiac troponin T.

The median concentrations of hs-cTnT and NT-proBNP at baseline were 4.29 (Q1-Q3: 2.99-7.25) pg/mL and 50.41 (Q1-Q3: 22.76-102.90) pg/mL, respectively. The incidence of all cancers increased with higher hs-cTnT levels across each endpoint (Figure 2A and Supplemental Table 1). Compared to participants with hs-cTnT levels below the LOD, those in the highest category (hs-cTnT ≥8.80 ng/L) showed a 2.8-fold increase in the incidence rate of all cancers. While there was a clear positive trend between higher hs-cTnT levels and increased incidence rates of cancer subtypes within the 3 highest categories, this trend was not consistently observed across all hs-cTnT categories, especially in the lower hs-cTnT categories, possibly due to relatively low incidence rate in these lower levels.

Dividing NT-proBNP into groups defined by quartiles, we observed generally higher incidence rates with elevated NT-proBNP levels. Participants in the highest NT-proBNP category (≥102.9 ng/L) experienced a 2.1-fold increase in the incidence rate of all cancers compared to those in the lowest quartile (<22.8 ng/L). However, an exception was breast cancer, where the group with NT-proBNP levels between 50.4 and 102.9 ng/L had a substantially lower incidence rate compared to the first 2 quartiles (Figure 2B, Supplemental Table 2), a pattern also reflected in female-specific cancers.

### Minimally adjusted and multivariate-adjustable cox proportional hazards models by hs-cTnT and NT-proBNP

We utilized Cox proportional hazards models to study the association between biomarkers hs-cTnT and NT-proBNP and the hazard of each specific cancer endpoint mentioned earlier. When testing for the proportionality of hazards, there was evidence to suggest the proportional hazards assumption was violated in the model considering NT-proBNP for the all-cancer endpoint (test of proportionality, *P* = 0.008 minimally adjusted and *P* = 0.018 fully adjusted). Consequently, for this model, we allowed the coefficient for BNP to depend on time. These results indicate that the positive association between baseline BNP and incident cancer decreases over time.

**Figure 3:**
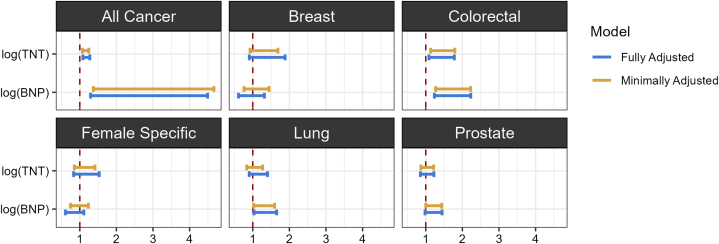
HRs With 95% CIs for hs-cTnT and NT-proBNP Levels for Cancer Endpoints in Adjusted Models HRs for the 2 primary predictors—log concentrations of hs-cTnT (TNT) and NT-proBNP (BNP) levels for each cancer endpoint were calculated with minimally adjusted (yellow lines) and fully adjusted (blue lines) models. The HRs were derived from the SDs for continuous covariates after standardization. Variables for minimally adjusted models: age, race/ethnicity, study site, sex, BMI, education, and health insurance status. Variables for fully adjusted models: all the covariates in the minimally adjusted plus diabetes, hypertension medication, systolic blood pressure, total cholesterol, HDL-C, smoking status, pack years of cigarette smoking, statins, and estimated glomerular filtration rate. BNP = N-terminal pro-B-type natriuretic peptide; HDL-C = high-density lipoprotein cholesterol; TNT = high-sensitivity cardiac troponin T; other abbreviation as in Figure 1.

As shown in Figure 3 and Table 4, for the all-cancer endpoints, the HRs of hs-cTnT and NT-proBNP were statistically significant in both minimally adjusted (HR: 1.16; 95% CI: 1.07-1.25; *P* < 0.001; and HR: 2.53; 95% CI: 1.37-4.67; *P* = 0.003, respectively) and fully adjusted models (HR: 1.18; 95% CI: 1.09-1.27; *P* < 0.001; HR: 2.41; 95% CI: 1.30-4.49; *P* = 0.006, respectively). Similar findings were observed for the colorectal endpoints with the HRs of hs-cTnT and NT-proBNP in the minimally adjusted (HR: 1.43; 95% CI: 1.13-1.80; *P* = 0.002; HR: 1.68; 95% CI: 1.27-2.23; *P* < 0.001, respectively) and fully adjusted models (HR: 1.39; 95% CI: 1.09-1.78; *P* = 0.009; HR: 1.66; 95% CI: 1.24-2.23; *P* < 0.001, respectively). For the lung cancer endpoint, NT-proBNP remained statistically significant in both models (HR: 1.28; 95% CI: 1.02-1.60; *P* = 0.030; HR: 1.31; 95% CI: 1.03-1.65; *P* = 0.025, respectively), while hs-cTnT was not significant in either. For the prostate cancer endpoint, NT-proBNP was statistically significant in the minimally adjusted model (HR: 1.20; 95% CI: 1.00-1.44; *P* = 0.049) but not in the fully adjusted model, while hs-cTnT was not significant in either. For the breast cancer and female-specific cancer endpoints, none of the primary predictors were significant in either the minimally or the fully adjusted models.

**Table 4:** HRs for Primary Predictors

Endpoint	Variable	Minimally Adjusted	Fully Adjusted	*P* Value	
				Minimally Adjusted^a^	Fully Adjusted^b^
All cancer outcome	log (TNT)	1.155 (1.071, 1.246)	1.176 (1.086, 1.273)	<0.001	<0.001
	log (BNP)	2.528 (1.37, 4.665)	2.412 (1.295, 4.491)	0.003	0.006
Breast outcome	log (TNT)	1.25 (0.927, 1.686)	1.306 (0.906, 1.884)	0.144	0.153
	log (BNP)	1.048 (0.76, 1.445)	0.896 (0.61, 1.317)	0.776	0.577
Colorectal outcome	log (TNT)	1.426 (1.133, 1.795)	1.392 (1.087, 1.781)	0.002	0.009
	log (BNP)	1.683 (1.273, 2.225)	1.661 (1.237, 2.229)	<0.001	<0.001
Female-specific outcome	log (TNT)	1.096 (0.851, 1.413)	1.127 (0.83, 1.53)	0.477	0.443
	log (BNP)	0.96 (0.747, 1.234)	0.822 (0.608, 1.11)	0.751	0.201
Lung outcome	log (TNT)	1.03 (0.836, 1.269)	1.125 (0.903, 1.403)	0.783	0.293
	log (BNP)	1.279 (1.024, 1.596)	1.307 (1.034, 1.653)	0.030	0.025
Prostate outcome	log (TNT)	1.027 (0.868, 1.215)	1.022 (0.854, 1.223)	0.757	0.811
	log (BNP)	1.199 (1.001, 1.436)	1.19 (0.98, 1.444)	0.049	0.079

BNP = N-terminal pro-B-type natriuretic peptide; TNT = high-sensitivity cardiac troponin T.

a Variables for minimally adjusted models: age, race/ethnicity, study site, sex, BMI, education, and health insurance status.

b Variables for fully adjusted models: all the covariates in the minimally adjusted plus diabetes, hypertension medication, systolic blood pressure, total cholesterol, HDL-C, smoking status, pack years of cigarette smoking, statins, and estimated glomerular filtration rate.

### Differences by race/ethnicities, sex, and age in the association of hs-cTnT and NT-proBNP with cancer endpoints

The interaction between race/ethnicity and hs-cTnT was statistically significant for the breast cancer and female-specific cancer endpoint (*P* = 0.008 and *P* = 0.013, respectively) (Supplemental Table 3). However, no individual contrast was statistically significant, indicating that the overall interaction may be driven by small differences across groups. For NT-proBNP, none of the interaction terms were statistically significant. The likelihood ratio tests revealed that none of the interaction terms involving sex and age in the association of hs-cTnT and NT-proBNP with the hazard of all cancers and individual cancer types were statistically significant.

## Discussion

Our results demonstrate that higher baseline levels of hs-cTnT and NT-proBNP are strong predictors of an increased risk of developing incident cancer events in a prospective, multiethnic, community-based cohort free of clinical CVD, even after comprehensive multivariable adjustment. The association of baseline levels of cardiac biomarkers with incident cancer events was unaffected by sex and race/ethnicity. When considering individual cancer types and sex-specific cancers, baseline levels of both cardiac biomarkers were associated with an increased risk of colorectal cancer, while only NT-proBNP was linked to lung cancer. In contrast, none of the sex-specific cancers were associated with baseline levels of cardiac biomarkers.

Previous epidemiological studies have shown a strong association between cancer survivors and increased CVD risk.^10^^,^^34^, ^35^, ^36^ In the ARIC (Atherosclerosis Risk In Communities) study population, cancer survivors exhibited a 42% increase in age-adjusted incidence rates of CVD compared to noncancer participants.^10^ Even after adjusting for traditional CV risk factors, cancer survivors remained at higher risks for CVD, particularly heart failure and stroke.^10^ The mechanisms underlying the strong cancer-CVD association were believed to involve shared pathological pathways, such as inflammation and oxidative stress, genetic predisposition and clonal hematopoiesis, and cardiotoxicity from cancer treatments.^10^^,^^37^

The cross talk between CVD and cancer also suggests that patients with CVD might exhibit an increased risk of developing cancer.^6^ Indeed, animal models of myocardial infarction, heart failure, and early cardiac remodeling have provided evidence that CVD might promote cancer growth and metastasis.^21^, ^22^, ^23^ However, clinical studies investigating the association between pre-existing CVD and subsequent cancer risk have been predominantly derived from retrospective analyses.^38^ For instance, an analysis of Danish nationwide registers between 1997 and 2016 found similar 5-year incidence rates of new-onset cancer in patients with heart failure, with rates of 20.9 and 20.2 per 1,000 person-years in 1997 and 2016, respectively.^39^ Examination of the IBM Market Scan claims data, which included 27 million individuals without a prior cancer diagnosis and with a minimum of 36 months of follow-up, suggested that those with CVD, particularly atherosclerotic CVD, had an increased risk of developing cancer compared to those without CVD.^40^

Two recent studies have examined the relationship between cardiac biomarkers and cancer incidence with slightly different results.^22^^,^^26^ In the PREVEND study, elevated hs-cTnT and NT-proBNP levels were predictive of higher cancer risk.^22^ In contrast, an analysis combining data from both the FHS and PREVEND studies found that only elevated natriuretic peptide levels, but not high-sensitivity troponin levels, were associated with a higher risk of cancer.^26^ It should be noted that cardiac biomarkers were measured in the FHS Offspring study at Examination 6 (1995-1998),^26^ and therefore, participants might not be completely free of CVD after a 20-year follow-up period from the initial recruitment between 1971 and 1975. In addition, 2 different cardiac troponins were used—hs-cTnI in FHS and hs-cTnT in PREVEND.

Our findings in the MESA cohort are consistent with those reported in the PREVEND study in that higher baseline hs-cTnT and NT-proBNP levels among participant free of clinical CVD had increased risks for all cancers and colorectal cancer, independent of traditional CVD risk factors. Compared to the FHS and PREVEND studies,^22^^,^^26^ the racial and geographic diversity in the MESA cohort with participants from 4 racial/ethnic groups (non-Hispanic White, Black, Hispanic/Latino, and Chinese) and 6 different field centers across the United States might enhance the generalizability of these findings. Baseline cardiac biomarkers such as hs-cTnT and NT-proBNP have been shown to predict incident heart failure and pathological cardiac remodeling/fibrosis in individuals free of CVD^25^^,^^33^ and correlate with heart aging.^41^ These findings suggest even mild elevation in these cardiac biomarkers may indicate subclinical CVD. Higher circulating hs-cTnT levels, even within the normal measurement range, are associated with an increased risk for adverse cardiac remodeling/fibrosis,^25^ which may indicate a chronic inflammatory response, a pathological process commonly shared between cardiac remodeling and cancer.^10^^,^^37^

Circulating cardiac troponin T and troponin I have been shown to be related with different outcomes, for instance, cardiac troponin T is more likely linked to the risk of non-CVD mortality compared to troponin I.^42^ The ARIC study demonstrates that hs-TnI and hs-TnT provide complementary, rather than redundant, information for predicting CVD risk^43^ and are associated differently with changes in left ventricular structure and function in the older population.^44^ Interestingly, cTnT has been detected in colorectal cancer tissue and may contribute to the proliferation and migration of colorectal cancer cells.^45^^,^^46^ Further research is required to establish the association between colorectal tissue remodeling and troponin elevation. Mildly elevated hs-cTnT levels may indicate underlying subclinical tissue remodeling in both the heart and colorectal tissues and contribute to myocardial injury and colorectal cancer, independent from traditional CV risk factors.

Similarly, increased plasma BNP level may be correlated to inflammation in cancer patients and a mouse model of colon cancer, in the absence of overt heart failure.^47^ Our findings also demonstrate a strong association between higher baseline NT-proBNP levels and an increased risk of lung cancer. Together with previous reports of elevated NT-proBNP levels in patients with lung cancer and acute exacerbation of chronic pulmonary diseases as well as the expression of BNP in human small cell lung cancer cells,^48^, ^49^, ^50^ these findings highlight the complex interaction between cardiovascular and pulmonary pathophysiology in the development of lung cancer.

Baseline levels of hs-cTnT and NT-proBNP were not associated with sex-specific cancers. Similarly, another CV risk marker independent of traditional CV risk factor,^51^ the coronary artery calcium score, has been shown to increase the risks for lung and colorectal cancers but not for sex-specific cancers in the MESA cohort.^32^ This lack of association with sex-specific cancers might be attributed to the distinct effects of hormonal processes on the cardiovascular and reproductive systems,^32^ such as estrogen, which can be protective against CVD^52^^,^^53^ but may increase the risk of sex-specific cancers like breast cancer.^54^

### Study limitations

Our study has several limitations. First, cancer data in the MESA cohort are recorded using ICD-9 codes during hospitalizations or inpatient procedures, which may lead to missed cancer diagnoses in participants who were never admitted for inpatient services.^51^ Second, there may be detection bias, as elevated baseline levels of hs-cTnT and NT-proBNP could lead to a higher incidence of CVD, which may result in more frequent hospitalizations or medical evaluations. Third, cancer events are not adjudicated by physicians, and we do not have data on cancer stage and treatment plans. Fourth, our study was limited by the small incidence rates of some cancer subtypes such as sex-specific cancers. Interpretation of the findings in Table 4 should proceed with caution given that no correction was done for multiple testing. Fifth, the MESA participants, with an average age of 62.3 years old, were free of CVD and serious medical conditions at enrollment. They were likely healthier than the general U.S. population of similar age. Finally, a single observational study, such as the one presented here, does not establish causality between higher hs-cTnT and NT-proBNP levels and the development of incident cancers.

## Conclusions

In the multiethnic adult MESA cohort free of clinical CVD and cancer at baseline, higher baseline levels of hs-cTnT and NT-proBNP were associated with a higher incidence rate of cancers over a 17.8-year follow-up period (Central Illustration). These findings suggest that hs-cTnT and NT-proBNP levels are not only indictive of cardiac events and mortality in cancer patients^17^, ^18^, ^19^, ^20^ but also predictive of future incident cancer risk in individuals with subclinical CVD.

Perspectives **COMPETENCY IN MEDICAL KNOWLEDGE:** Higher baseline levels of hs-cTnT and NT-proBNP are strong predictors of an increased risk of developing incident cancer events, independent of traditional cardiovascular risk factors. These associations between hs-cTnT and NT-proBNP levels and incident cancer events are not affected by sex and race/ethnicity.

**TRANSLATIONAL OUTLOOK:** Our findings suggest that levels of hs-cTnT and NT-proBNP are not only indictive of cardiovascular events and mortality in cancer patients but also predictive of future incident cancer risk in individuals with subclinical CVD.

**Central Illustration:**
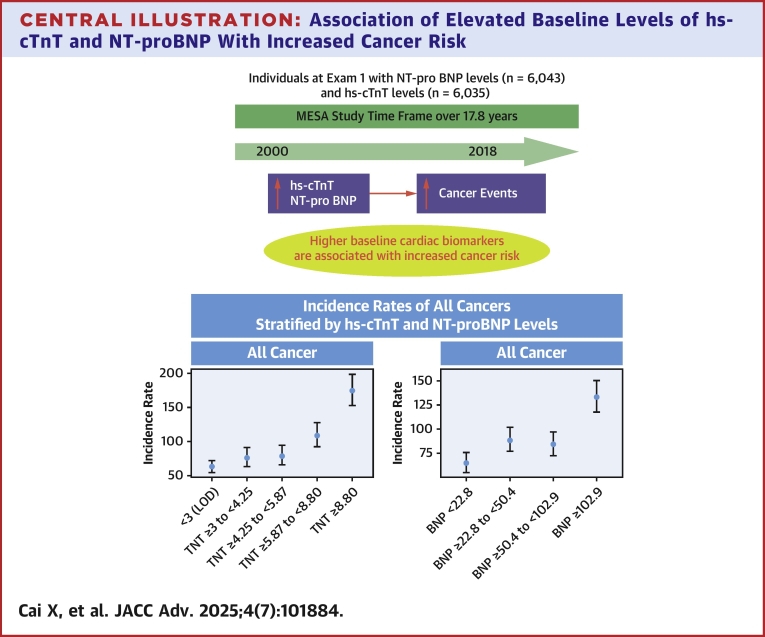
Association of Elevated Baseline Levels of hs-cTnT and NT-proBNP With Increased Cancer Risk In the multiethnic MESA cohort, higher baseline levels of the cardiac biomarkers hs-cTnT and NT-proBNP, representing subclinical cardiovascular disease, were associated with an increased risk of incident cancer over a median follow-up period of 17.8 years, as demonstrated by incidence rates of all cancers stratified by hs-cTnT (TNT) and NT-proBNP (BNP) levels. hs-cTnT = high-sensitivity cardiac troponin T; LOD = limit of detection; MESA = Multi-Ethnic Study of Atherosclerosis; NT-proBNP = N-terminal Pro-B-type Natriuretic Peptide.

## Funding support and author disclosures

The MESA study was supported by the 10.13039/100000050National Heart, Lung, and Blood Institute (contracts 75N92020D00001, HHSN268201500003I, N01-HC-95159, 75N92020D00005, N01-HC-95160, 75N92020D00002, N01-HC-95161, 75N92020D00003, N01-HC-95162, 75N92020D00006, N01-HC-95163, 75N92020D00004, N01-HC-95164, 75N92020D00007, N01-HC-95165, N01-HC-95166, N01-HC-95167, N01-HC-95168, N01-HC-95169); and the 10.13039/100006108National Center for Advancing Translational Sciences (grants UL1-TR-000040, UL1-TR-001079, UL1-TR-001420). Dr Cai is supported, in part, by a Mentored Clinical Scientist Career Development Award (HL168147) from the 10.13039/100000050National Heart, Lung, and Blood Institute. Dr Yang has received research funding from 10.13039/100008322CSL Behring, Boehringer Ingelheim and Eli and Lilly, Amgen, Janssen Research and Development, and Bristol Myers Squibb (nonrelevant to the current study), and consulting fees from Pfizer, Xencor, and Edwards Lifesciences (nonrelevant to the current study). Dr deFilippi reports consulting for Abbott Diagnostics, FujiRebio, Quidel:Ortho, Roche Diagnostics, Tosoh, and Siemens Diagnostics (nonrelevant to the current study). All other authors have reported that they have no relationships relevant to the contents of this paper to disclose.

## Footnotes

Appendix For supplemental methods, tables, and a figure, please see the online version of this paper.
