# Supplementary material for: Baseline Cardiac Biomarker Levels as Predictors of Cancer Risk in the MESA Cohort
Source: JACC Adv. 2025 Jun 16;4(7):101884. doi: 10.1016/j.jacadv.2025.101884 (PMC12213292; doi:10.1016/j.jacadv.2025.101884)
Supplement: Supplementary data [file mmc1.docx]

**SUPPLEMENTAL APPENDIX**

**Supplemental Methods**

**Baseline Characteristics**

Dietary patterns were evaluated using a self-administered, 120-item food frequency questionnaire.^1,2^ The diet metric includes five factors: intake of fruits and vegetables, fish, sodium, sugar-sweetened beverages, and whole grains. Participants were categorized as having a poor or not poor diet based on meeting 0-1 or 2-5 intake recommendations, respectively.^3^ Diabetes mellitus was defined by a fasting plasma glucose level ≥ 126 mg/dL or the use of antidiabetic medications.^4^ Hypertension was defined as systolic blood pressure ≥ 140 mmHg, diastolic blood pressure ≥ 90 mmHg, or the use of antihypertensive medications. Smoking status was evaluated through a self-completed questionnaire as previously reported.^5^ Participants were categorized as current smokers, former smokers, or non-smokers. For current or former smokers, smoking history was quantified in pack-years. Physical activity was assessed using a semiquantitative survey adapted from the Typical Week Physical Activity Survey, as previously described.^6^.

**Biomarker Assays**

Hs-cTnT and NT-proBNP levels were measured in plasma samples collected from participants at baseline at MESA Exam 1 (2000-2002). The samples were then frozen at -70 °C and stored until measurement of cardiac biomarkers at later dates.^7,8^ Hs-cTnT levels were determined using the Cobas e601 analyzer (Roche Diagnostics, Indianapolis, IN) at the University of Maryland.^7^ The interassay coefficients of variation in the MESA cohort were reported as 3.6% at 28 ng/L and 2.0% at 2154 ng/L, with a detection limit of 3 ng/L.^7^ NT-proBNP levels were measured using a highly sensitive and specific Elecsys electrochemiluminescence immunoassay (Roche Diagnostics, Indianapolis, IN), which employs the double-antibody sandwich method, at the University of California, San Diego.^9^ The intra- and interassay coefficients of variation for different NT-proBNP levels were as follows: 2.7% and 3.2% at 175 pg/mL, 2.4% and 2.9% at 355 pg/mL, 1.9% and 2.6% at 1068 pg/mL, and 1.8% and 2.3% at 4962 pg/mL, respectively.^9^

**References:**

1. Nettleton JA, Schulze MB, Jiang R, Jenny NS, Burke GL, Jacobs DR, Jr. A priori-defined dietary patterns and markers of cardiovascular disease risk in the Multi-Ethnic Study of Atherosclerosis (MESA). *Am J Clin Nutr*. 2008;88:185-194. <https://doi.org/10.1093/ajcn/88.1.185>

2. Nettleton JA, Polak JF, Tracy R, Burke GL, Jacobs DR, Jr. Dietary patterns and incident cardiovascular disease in the Multi-Ethnic Study of Atherosclerosis. *Am J Clin Nutr*. 2009;90:647-654. <https://doi.org/10.3945/ajcn.2009.27597>

3. Cai X, White Q, Wang DR, et al. Cardiovascular Risks and Outcomes Among Chinese American Immigrants: Insights From the Multi-Ethnic Study of Atherosclerosis. *J Am Heart Assoc*. 2024:e037114. <https://doi.org/10.1161/JAHA.124.037114>

4. Bertoni AG, Kramer H, Watson K, Post WS. Diabetes: Insights from the Multi-Ethnic Study of Atherosclerosis. *Glob Heart*. 2016;11:337-342.

5. Al Rifai M, DeFilippis AP, McEvoy JW, et al. The relationship between smoking intensity and subclinical cardiovascular injury: The Multi-Ethnic Study of Atherosclerosis (MESA). *Atherosclerosis*. 2017;258:119-130. <https://doi.org/10.1016/j.atherosclerosis.2017.01.021>

6. Bertoni AG, Whitt-Glover MC, Chung H, et al. The association between physical activity and subclinical atherosclerosis: the Multi-Ethnic Study of Atherosclerosis. *Am J Epidemiol*. 2009;169:444-454. <https://doi.org/10.1093/aje/kwn350>

7. Seliger SL, Hong SN, Christenson RH, et al. High-Sensitive Cardiac Troponin T as an Early Biochemical Signature for Clinical and Subclinical Heart Failure: MESA (Multi-Ethnic Study of Atherosclerosis). *Circulation*. 2017;135:1494-1505. <https://doi.org/10.1161/CIRCULATIONAHA.116.025505>

8. Daniels LB, Clopton P, deFilippi CR, et al. Serial measurement of N-terminal pro-B-type natriuretic peptide and cardiac troponin T for cardiovascular disease risk assessment in the Multi-Ethnic Study of Atherosclerosis (MESA). *Am Heart J*. 2015;170:1170-1183. <https://doi.org/10.1016/j.ahj.2015.09.010>

9. Choi EY, Bahrami H, Wu CO, et al. N-terminal pro-B-type natriuretic peptide, left ventricular mass, and incident heart failure: Multi-Ethnic Study of Atherosclerosis. *Circ Heart Fail*. 2012;5:727-734. <https://doi.org/10.1161/CIRCHEARTFAILURE.112.968701>

**Supplemental Figure 1. Study Flow Chart**

The flow chart illustrates the inclusion and exclusion criteria for hs-cTnT (*A*) and NT-proBNP (*B*) among MESA participants, based on the availability of covariates and biomarker measurements.


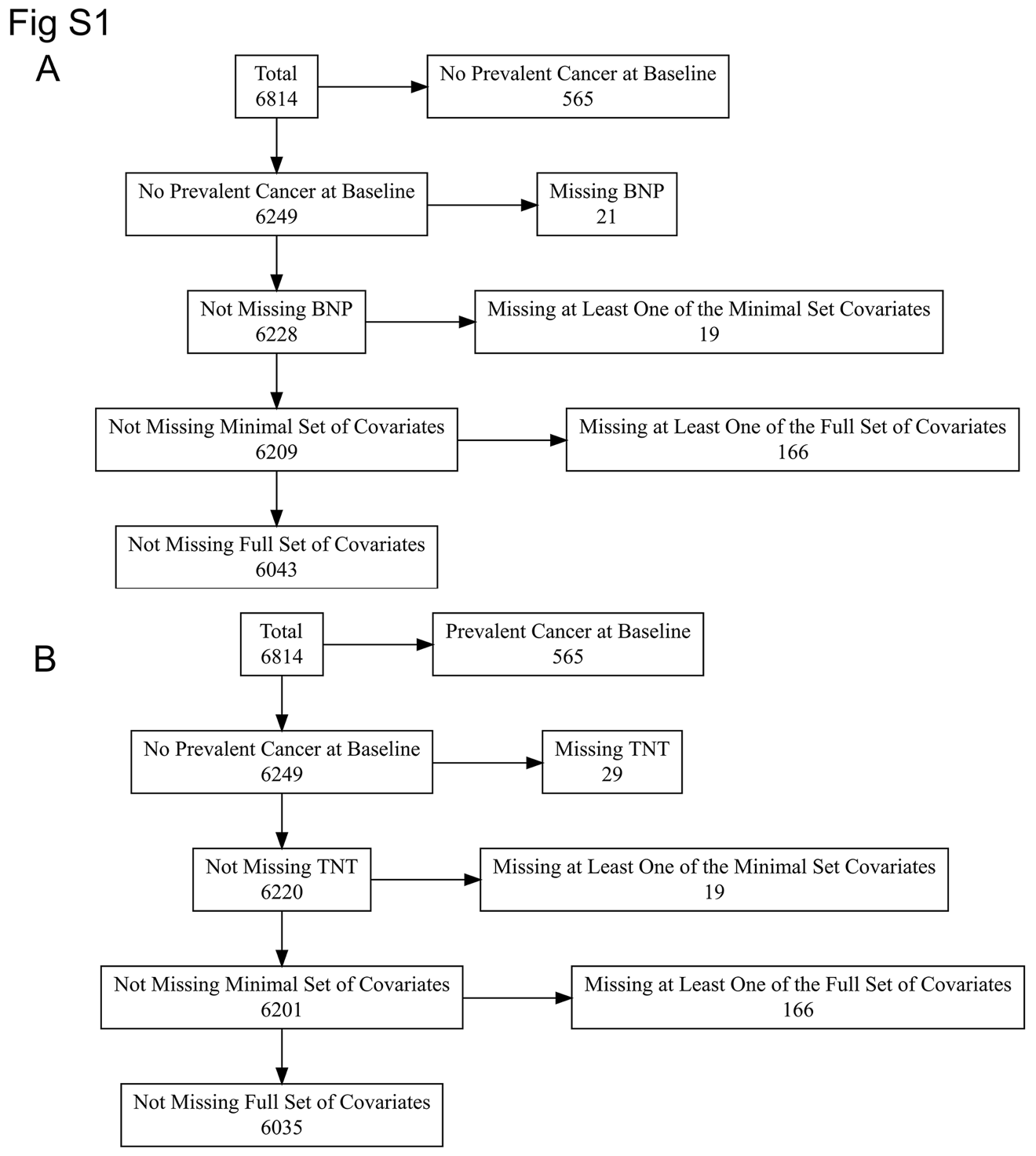


**Supplemental Table 1: Incidence Rates^1^, Stratified by hs-cTnT (TNT) Levels**

| **Cancer** | **< 3 (LOD)**  **(n=2043)** | **3 <= TNT < 4.25 (n=1023)** | **4.25 <= TNT < 5.87 (n=1021)** | **5.87 <= TNT < 8.80 (n=1011)** | **TNT >= 8.80**  **(n=1117)** |
| --- | --- | --- | --- | --- | --- |
| All Cancer | 62.69  (54.26, 72.05), n=198 | 76.17 (63, 91.29),  n=117 | 79.02  (65.56, 94.41),  n=121 | 108.91  (92.39, 127.54), n=154 | 174.54  (152.66, 198.67),  n=229 |
| Prostate | 31.24 (20.41, 45.78),  n=26 | 20.46 (11.19, 34.33),  n=14 | 38.9  (26.25, 55.54), n=30 | 41.18  (28.84, 57.02), n=36 | 53.31 (39.69, 70.09), n=51 |
| Female Specific | 18.05  (13.01, 24.4),  n=42 | 17.61  (9.86, 29.05),  n=15 | 17.1  (9.11, 29.24), n=13 | 22.23 (11.49, 38.83), n=12 | 39.4  (21.54, 66.11), n=14 |
| Breast | 11.18  (7.3, 16.38),  n=26 | 9.39 (4.05, 18.51),  n=8 | 10.52  (4.54, 20.73),  n=8 | 20.38  (10.17, 36.46), n=11 | 22.52  (9.72, 44.37),  n=8 |
| Lung | 11.4 (7.98, 15.78), n=36 | 8.46  (4.51, 14.47),  n=13 | 9.8  (5.48, 16.16),  n=15 | 15.56  (9.75, 23.56),  n=22 | 22.87  (15.43, 32.64), n=30 |
| Colorectal | 3.48 (1.74, 6.23), n=11 | 8.46 (4.51, 14.47),  n=13 | 5.22  (2.26, 10.29),  n=8 | 7.78  (3.88, 13.92),  n=11 | 22.1  (14.8, 31.74),  n=29 |

^1^Incidence rates were presented per 10,000 person-years with confidence intervals assuming a Poisson distribution of the event counts. The strata were selected based on previous work.^7^

**Supplemental Table 2: Incidence Rates^1^, Stratified by NT-pro BNP (BNP) Levels**

| **Cancer** | **BNP < 22.8 (n=1555)** | **22.8 <= BNP < 50.4 (n=1556)** | **50.4 <= BNP < 102.9 (n=1555)** | **BNP >= 102.9 (n=1557)** |
| --- | --- | --- | --- | --- |
| All Cancer | 64.96  (55.2, 75.95), n=157 | 89.14 (77.41, 102.15), n=207 | 84.44  (72.86, 97.34),  n=190 | 133.4  (117.8, 150.51),  n=264 |
| Prostate | 23.54  (16.74, 32.18), n=39 | 36.55 (26.23, 49.59),  n=41 | 51.12  (36.84, 69.1),  n=42 | 65.89  (45.9, 91.64),  n=35 |
| Female Specific | 18.42  (10.07, 30.9), n=14 | 21.66 (14.15, 31.74),  n=26 | 14  (8.55, 21.62),  n=20 | 24.87  (17.42, 34.42),  n=36 |
| Lung | 7.86  (4.73, 12.28), n=19 | 11.2  (7.31, 16.41),  n=26 | 12.89  (8.63, 18.51),  n=29 | 21.22  (15.3, 28.69),  n=42 |
| Breast | 13.16  (6.31, 24.2), n=10 | 13.33  (7.62, 21.64),  n=16 | 7 (3.36, 12.87),  n=10 | 17.27  (11.17, 25.49), n=25 |
| Colorectal | 2.9  (1.16, 5.97), n=7 | 6.46  (3.62, 10.65),  n=15 | 8  (4.74, 12.64),  n=18 | 16.17 (11.06, 22.83),  n=32 |

^1^Incidence rates were presented per 10,000 person-years with confidence intervals assuming a Poisson distribution of the event counts. The strata for NT-proBNP were defined based on the quartiles of NT-proBNP.

**Supplemental Table 3: Models Including the Interaction Between Race/Ethnicity and Each Primary Predictor**

| **Endpoint** | **LR Test P-Value** | **Variable** | **Interaction** | **Interaction P-Value** | **Main + Interaction** | **Main** |
| --- | --- | --- | --- | --- | --- | --- |
| **log(TNT)** | | | | | | |
| Breast | 0.008 (*) | Black: log(TNT) | 0.63 (0.32, 1.25) | 0.19 | 1.24 (0.74, 2.07) | 1.98 (1.18, 3.30) |
| Female Specific | 0.013 (*) | Black: log(TNT) | 0.64 (0.35, 1.14) | 0.13 | 1.02 (0.64, 1.61) | 1.60 (1.07, 2.41) |
|  |  | Hispanic/ Latino:log(TNT) | 0.33 (0.10, 1.04) | 0.059 | 0.53 (0.17, 1.59) |  |

Hazard ratios with 95% CIs were presented in the table.
